# Semi-automated modeling of reaction states in time-resolved serial femtosecond crystallography using molecular dynamics sampled conformations

**DOI:** 10.1063/4.0000779

**Published:** 2025-10-31

**Authors:** Sriram Srinivasa Raghavan, Keiko Shinoda, Eriko Nango, Florence Tama, Osamu Miyashita

**Affiliations:** 1 RIKEN Center for Computational Science, Kobe 650-0047, Japan; 2 Research Center for Materials Informatics, Department of Advanced Data Science, The Institute of Statistical Mathematics, Tachikawa, Tokyo 190-8562, Japan; 3 Institute of Multidisciplinary Research for Advanced Materials, Tohoku University, Sendai 980-8577, Japan; 4 Department of Physics, Graduate School of Science, Nagoya University, Nagoya 464-8601, Japan; 5 Institute of Transformative Bio-Molecules, Nagoya University, Nagoya 464-8601, Japan

## Abstract

Time-resolved serial femtosecond crystallography (TR-SFX) is a technique designed to reveal the molecular dynamics underlying chemical reactions, thereby providing insights into the relationship between structure and function. By capturing a series of conformational changes in intermediate states, TR-SFX enables the visualization of dynamic structural transitions. In this study, we have presented a new approach for modeling reaction state conformations using molecular dynamics (MD) simulations. In this approach, MD simulations were first performed to generate a large number of conformational samples, which were then used as initial models for refinement against diffraction data from the triggered states, thereby facilitating the construction of accurate dynamic structure models. The derived models were evaluated using tools such as Edstats and MolProbity to identify models with high-quality geometries and local electron density metrics. This procedure provides a semi-automated approach for building dynamic structural models from TR-SFX data, ensuring their robustness for further exploration and understanding of macromolecular dynamics.

## INTRODUCTION

Time-resolved serial femtosecond crystallography (TR-SFX) is an advanced structural biology technique that combines the ultrafast imaging capability of serial femtosecond crystallography using x-ray free electron lasers with time-resolved experiments. This approach provides detailed structural insights into ultrafast biochemical and biophysical processes that occur within biological macromolecules.^1,2^ TR-SFX experiments employ the “pump-probe” technique to capture a series of structural snapshots of proteins to create a “molecular movie” that reveals the structural dynamics during biological processes.^3,4^

The data analysis of TR-SFX consists of several procedures, including the initial data preprocessing of large raw diffraction patterns^5^ using tools such as CrystFEL^6^ or cctbx.xfel.^7^ The next stage of TR-SFX data processing involves extracting a small structural difference in the structure-factor amplitudes between the triggered- and reference-state datasets. The analysis of diffraction data from reaction-triggered crystal systems often reveals that a large portion of the structure remains similar to that of the reference state, with only a certain fraction of the molecules transitioning to the triggered state.^8–10^ Because reference-state populations are dominant, refinement algorithms often return a structure similar to the reference state when refinement is performed against a triggered-state dataset.^9^ In order to address this issue, a common approach is to observe meaningful difference signals of the structural factors by examining isomorphous difference density maps between the triggered-state and reference-state data.^11,12^ Electron density features in isomorphous difference maps can be used to interpret the associated changes, where the atoms moved from the reference model appear negative in electron density,^8^ which is typically used as guidance for modeling the structure.

Model refinements have been performed in the past using a few different approaches. First, one could refine the model against the extrapolated structure factor of the triggered state. The concept of structure-factor extrapolation technique was first introduced by Genick *et al.*^11,12^ However, there is a substantial variation in the methods used to estimate the extent of structure-factor extrapolation. These variations include differences in the choice of the residue mask applied to the difference density map, which defines the region considered for the occupancy/density estimations, such as around the chromophore in the photoactivated yellow protein^13^ or covering all of the difference density peaks above a certain Z-score in the protein.^14^ There are also variations in whether the experimentally measured structure factor for the reference state or the structure factor calculated from the reference model is used to estimate the extrapolated structure factor.^1,11,15^ In general, the R-factors associated with the structure refined in the extrapolated map are higher than those in the conventional model.^16^ This was due to the exaggeration of the associated experimental errors obtained from the extrapolation methods.^16^ In addition, it is challenging to estimate the true population of a triggered state.^15^

Alternatively, the triggered-state structure can be modeled using a multiconformational model to interpret the triggered-state dataset. A reference-state model is used as the fixed main structure, and the triggered state is modeled as an alternative conformation, which is refined against the triggered-state diffraction data. A variation of this protocol, established by Neutze *et al.*, focused on modeling the region constituting the triggered-state residue, as identified from the isomorphous difference density map.^17,18^

To describe a triggered state using a two-state conformational model, a challenge in traditional protein crystallography must be considered. Typically, a protein crystal structure is represented by a single set of atomic coordinates corresponding to the mean positions of atoms across the crystal. If the protein conformation includes discrete atomic configurations associated with alternative positions, each corresponding to a distinct conformer with different minima in the free-energy landscape, the positional variance is captured by the temperature factor (B-factor), which often differs from that of the neighboring region. In such cases, an alternative atomic conformation with partial occupancy describes the fraction of crystals with similar B-factors. In addition, when structural models based on a few conformational representations are inadequate for capturing the conformational variations present in protein crystals, other approaches can be explored, including modeling selected side chains with multiple conformers using tools such as Ringer,^19^ FLEXR,^20^ and qFit3,^21^ or using restrained molecular dynamics.^22^

Another approach, *ensemble refinement* combines structure refinement with molecular dynamics (MD) guided by diffraction data to produce ensemble models fitted to diffraction data.^23–26^ In ensemble refinement, small-scale local molecular vibrations and the anharmonic motions of individual atoms are captured by MD simulations that generate snapshots of different conformations. These MD-generated snapshots collectively sample a wider range of local flexibilities, extending beyond simple harmonic models. Meanwhile, the broader, collective domain movements (anisotropic disorder) are modeled using the translation–libration–screw (TLS) approach, which describes large-scale translational and rotational motions. By combining these methods, ensemble refinement can represent both anisotropic (directional) and anharmonic (nonlinear) disorders and is implemented in Phenix as *phenix.ensemble_refinement.*^23,27^ The ensemble refinement procedure can elucidate the inherent flexibility of a region and is potentially useful for capturing conformations in the triggered state.

In addition to ensemble refinement, MD simulations have been employed to provide better interpretations of x-ray crystallographic experimental data. Here, crystalline MD simulations^28^ are often used, where proteins are simulated in crystal environments, for better comparison to experimental data. For example, MD-MX method^29^ performs crystalline MD to generate structure factors and electron density maps that can be directly compared with experimental diffraction data, enabling validation of models. Crystal simulations are also often used to probe the origin of diffuse scattering,^30,31^ where MD trajectories provide insights into correlated atomic motions that contribute to diffuse intensity. Crystal MD simulations were also employed to investigate water dynamics and hydrogen-bond networks in Photosystem II, by directly comparing simulated electron densities with experimental XFEL densities.^32^

In the context of model-building tasks from crystallographic data, the AMBER forcefield can be used to maintain the geometry of molecules in the structure refinement^28,33–36^ and quantum mechanical refinement methods^37–40^ are also available in modern crystallographic software. Furthermore, AMBER MD simulation engine has been recently utilized to enhance structure refinement approaches.^35–36^ These new approaches provide alternative strategies for incorporating physical principles and dynamic information into structural models.

The studies discussed above have substantially advanced the integration of MD with crystallographic modeling, yet they are primarily focusing on steady-state diffraction problems. In contrast, as discussed above, the analysis of TR-SFX data poses other challenges, as one must resolve very low-occupancy, transient intermediates whose signals are frequently overwhelmed by resting-state density. Smith *et al.*, for example, used the differences in the electron densities between two MD simulations to compare with the experimental difference map data to validate structural hypotheses in catalysis.^41^

In this study, we explore other possibilities of utilizing MD simulations to automate two-state conformer modeling process for TR-SFX data. We use MD simulations to sample physically plausible conformer candidates, which are then employed in multiconformer refinement with occupancy- and geometry-aware validations to derive models that describe weakly populated reaction states. This strategy provides an alternative approach to reducing the reliance on manual building, a challenge in time-resolved crystallography.

As an implementation of this approach, 4000 MD frames representing distinct conformational snapshots of the protein were used as starting points for the refinement runs. These conformations were then refined against the structural factors of the triggered state as a two-state model using the fixed reference-state structure and MD-derived conformers as inputs for triggered-state structural refinement using Phenix.^27^ Model refinement was achieved through the adjustment of model parameters, such as atomic coordinates and occupancy, in an iterative process to compare the fit of the obtained model with experimental data. The final model was validated using global metrics such as R-work and R-free.^41–45^ It is also important to validate the local structure of the model that accurately reflects the experimental data.^46–48^ In order to ensure model accuracy and precision, we have employed various electron density metrics, such as the real-space difference score (RSZD), to assess the significant difference in the density peaks around, and the real-space Z-observed (RSZO) score to estimate the signal-to-noise ratio of the density in the region.^49^ Additionally, the real-space correlation coefficient^49^ (RSCC) was used to validate the refined models, and further geometric validation was performed using MolProbity.^50^

We evaluated the performance of this model-building approach using bacteriorhodopsin (bR) data previously published by Nango *et al.*^17^ The aim was to determine whether numerous refinement trials from a large set of initial models sampled from MD trajectories could yield a better model than manually fitted models that also closely resembled the isomorphous difference density features. The obtained results indicate that the proposed pipeline, which combines MD-based sampling and structure refinement calculations, can assist the model-building process from TR-SFX data. By providing a more automated protocol that can reduce reliance on manual fitting, the proposed approach could broaden access to time-resolved crystallography for researchers.

## MATERIALS AND METHODS

### Protocol overview

**FIG. 1.**
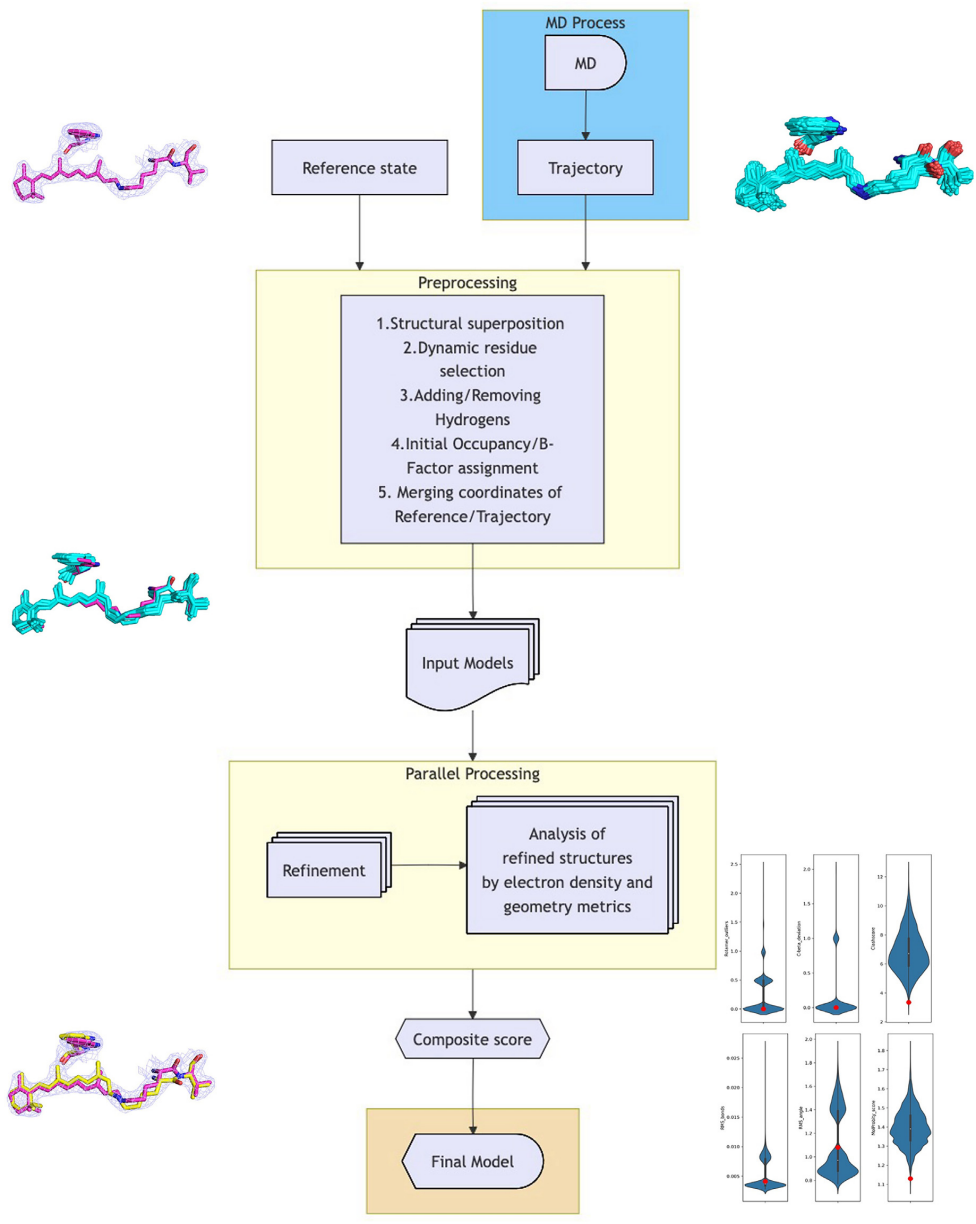
Workflow of the algorithm. The process performed two-state conformational modeling of the triggered state. The procedure was initiated by generating input models from MD trajectories. Structural superposition with the reference structure was first performed, and triggered-state residues were selected and combined with the reference structure as alternative conformations. Subsequently, B-factor and initial occupancy assignments and the addition of hydrogens were performed. It then executed *phenix.refine* in parallel to refine the coordinates and group occupancies for triggered-state conformations.^27^ The final models were selected based on the lowest composite score.

We propose an approach that utilizes conformational sampling via MD simulations to improve structural model building using TR-SFX crystallographic data. First, the MD frames are aligned with the reference-state structure. Conformer samples of the residues identified as possessing alternative conformations from the difference density maps were extracted from the MD frames and merged with the reference state as alternative conformers. The B-factors are set to match the reference state. These merged structures were subsequently used as the initial starting model for the coordinate and group occupancy refinement of the alternative conformer against the triggered-state dataset. The workflow associated with this method is illustrated in Fig. 1.

### Target system

We have demonstrated the proposed approach using data from TR-SFX experiments performed on bacteriorhodopsin (bR) by Nango *et al.*^17^ Their study aimed to visualize conformational changes in bR occurring between 16 ns and 1.725 ms following photoactivation. Their results revealed that proton transfer from the Schiff base (SB) to Asp 85 induces structural changes in the retinal. Specifically, the changes are modeled as a displacement of the SB toward the cytoplasmic (CP) side by 0.15 Å and a shift of the C20 methyl group by 0.21 Å within the time interval 760 ns ≤ Δt ≤ 36.2 *μ*s.^17^ Additionally, a flip in the carbonyl tail of Lys 216 was observed at all recorded time points except for the 16 ns structure. The published structure of the resting-state (PDB: 5B6V) was used as the reference state (A conformer). To model the triggered state (B-conformer), the initial models used for refinement were derived from MD simulations.

### MD simulation to generate sample conformations

The initial coordinates of bR (earliest state after excitation, 16 ns) were obtained from PDB 5B6W.^17^ Archaeal lipid bilayers were built using the Memgen program^51^ and consisted of 347 molecules of 2,3-di-O-phytanyl-sn-glycerol-1-phosphatidylethanolamine and 345 molecules of 2,3-di-O-pentanoyl-sn-glycerol-1-phosphatidylethanolamine. The lipid molecules surrounding the bacteriorhodopsin were modeled based on the partially resolved lipid structures observed in the crystal structures. The membrane was pre-equilibrated for 200 ns before embedding. The bacteriorhodopsin was embedded in a pre-equilibrated membrane using LAMBADA.^52^ Overlapping lipid molecules whose heavy atoms approached within 0.12 nm of either bacteriorhodopsin or the modeled lipids were removed.

MD simulations were performed using GROMACS Version 2018.4.^53^ The all-atom Fuji force field^54–56^ was used for all components of the system, including proteins, lipids, the retinal moiety, and ions, combined with explicit TIP3P water.^57^ Partial atomic charges of the retinal chromophores were determined by fitting the electrostatic potential using the RESP procedure.^58^ Protonation states were assigned by the GROMACS *pdb2gmx* tool. Asp96, Asp115, and Glu204 were protonated, and the Lys-retinal Schiff base was modeled in its protonated state. The systems were solvated using GROMACS *solvate*, without retaining crystallographic waters; however, in the course of MD, water molecules occupied positions close to those near the chromophore observed in the crystal structure. All systems were hydrated with a 150 mM NaCl electrolyte. The temperature was set to a constant value (298 K) using a Nosé–Hoover thermostat with a coupling constant of 1.0.^59–60^ The pressure was set to 1 bar using a semi-isotropic Berendsen barostat^61^ or a Parrinello–Rahman barostat,^62^ with a coupling constant of 2.0 ps for both barostats. Electrostatic interactions were calculated using the particle mesh Ewald (PME) method^63^ with a real-space cutoff of 1.0 nm. The Lennard–Jones interactions were calculated using the Lennard–Jones (LJ) PME (LJ-PME) method^64^ with geometric approximations of the combination rules in the reciprocal space. The Verlet cutoff scheme was used for the neighbor list. The linear constraint solver (LINCS) algorithm^65^ with a LINCS order of six was used to constrain all of the bonds. A virtual site model was adopted to remove the bond angle degrees of freedom from the hydrogen atoms.

System energy was minimized using alternating steepest-descent and conjugate gradient methods, followed by isothermal-isobaric ensemble equilibration using the semi-isotropic Berendsen barostat, with position restraints only on the heavy atoms of the protein for 30 ns with a time step of 2 fs. Then, unconstrained simulations were performed using a semi-isotropic Berendsen barostat, initially for 20 ns with a time step of 2 fs, and later for 10 ns with a time step of 5 fs. Using the final structures as the initial structure, three unbiased simulations were carried out with a time step of 5 fs, using a semi-isotropic Parrinello–Rahman barostat with a different initial velocity that satisfied a Maxwell–Boltzmann distribution at 298 K. Production runs were performed for 2 *μ*s for the state at 16 ns after excitation. Snapshots for analysis were extracted every 500 ps throughout the production runs, yielding 4000 conformations.

### Generation and refinement of the MD-based models

MD trajectories of bR corresponding to the triggered state, containing the *cis* configuration of the retinal chromophore, spanned 2 *μ*s and comprised 4000 frames. The first 200 frames were not used in the analysis to eliminate the influence of the x-ray structure, which was used as the initial conformation for the MD simulation. The conformers derived from the MD trajectory frames were aligned to the reference-state structure (PDB: 5B6V) based on the Cα atoms using PyMOL.^66^ Dynamic residues for which we attempted to construct a model of the triggered state, could be identified based on the presence of difference density in the isomorphous difference density map. In this study, we have selected dynamic residues identical to previously published structures for a specific timeframe for better comparison.^17^ The naming of nonhydrogen atoms in the MD-derived structures was made consistent with the reference crystal structure data. The selected residues were merged as alternative conformers with the reference (resting-state) structure using PyMOL.^66^ Hydrogen atoms removed from the trajectories were re-added using *phenix.reduce* to comply with the naming conventions of *phenix.refine* and facilitate further refinement.^27,67^ The addition of hydrogen was optional within the workflow in order to optimize computational efficiency, allowing this step to be deferred until the final refined model, if desired. However, in the reported procedure, hydrogen atoms were added to each input PDB file obtained from trajectory sampling before refinement, ensuring consistency and accuracy in the initial stages of the refinement process. The hydrogen atom positions were refined using a riding model.

A reduced dataset for various time series of TR-SFX measurements of bR, ranging from 16 ns to 1.725 ms, was used.^17^ The R_free_ flags, which specify the fraction of reflections used for the R_free_ calculations, were used, as in the original study. The bR structure includes a retinal chromophore (Ret 300) covalently bonded to Lys 216 in the helix G region.^17^ To properly establish the restraint of the covalent bond to Lys 216 via a Schiff base, *phenix.ready_set* was employed to add hydrogen atoms to the retinal chromophre.^27^ Additionally, *phenix.reduce* was configured with a nonmetal bump option set to −3.0 Å to enable the addition of three hydrogen atoms to the NZ atom of Lys 216 side chain.^17^ Subsequently, two hydrogen atoms were removed using cctbx atom selection to accommodate the covalent bond to the C15 atom of RET 300, ensuring that the proper valency is preserved in the merged structure.^68^ Finally, this definition was used in the refinement for the geometric restraint associated with the covalently bound retinal residue.^17^ The model was refined against the experimental structural factors using *phenix.refine*, adhering to version 1.10, which is the same version used in the original study.^17,27^

Coordinates and group occupancy refinement strategies were applied to the residues modeled as alternative conformers. Here, the B-factors of the selected residues were fixed to match those of the reference-state structure, assuming equivalent atomic displacements in both the reference and triggered systems. This approach was also employed in the original experimental study.^17^ In the initial input model, the occupancy of the B-conformer was set to 25%, subject to adjustments during group occupancy refinement. During refinement, all the atoms in the B-conformers were assigned to a single occupancy group, forcing them to refine to a common value throughout the structure. Occupancy of the A conformer was automatically maintained as the complement (1- occupancy B) for all residues corresponding to the triggered state.

The refinements were performed using the target weights (*wxc_scale*). Increasing the target weights in Phenix gives more weight to the x-ray data during refinement, resulting in a better fit to the experimental data.^27^ However, this also induced geometric distortions in the model, with greater deviations from the ideal bond lengths and angles. Balancing this parameter is essential for achieving an accurate geometry and a good fit to the data.^27^ The *wxc_scale* ranging from 0.02 to 3.0 were systematically tested, and a final weight of 0.035 was selected, as the refinement of the dark model at this weight resulted in the lowest R-factors among the tested weights. The refinement of conformation B, coupled with the group occupancy, was limited to three cycles of the reciprocal space refinement. This approach may restrict exploration of the conformational space during refinement and preserve the conformational heterogeneity obtained from the MD samples.

In addition to the refinement trials from MD sample conformers, for comparison, we also performed Phenix refinement of the triggered-state conformer using the published reference-state structure 5B6V as the initial conformer. These refinements were performed against the structural factors for various time-series datasets.

### Validation metrics for quantitative model comparison

The model was validated using triggered-state conformers based on the geometry and electron density fit of the model. The geometry was validated using MolProbity,^50^ which compares the input model with idealized values and provides component scores for various geometric and steric features, summarized by the overall MolProbity score.

Density-based metrics are calculated from *F*o-*F*c as well as 2*F*o-*F*c maps using the EDSTATS tool,^49^ including the real-space correlation coefficient (RSCC), real-space Z-difference score (RSZD), and real-space Z-observed score (RSZO). The ideal values for the RSCC, RSZD, and RSZO metrics followed those outlined by Pearce *et al.*^69^ The RSCC measures how well the model agrees with the experimental data, with values above 0.7, indicating a strong match. RSZD evaluates the accuracy of the model by examining the difference in density around a residue, where a score below three is desirable. The RSZO assesses the model's precision by averaging the electron density over a residue and dividing it by the map's noise, with a preferred value above 2.

### Distribution of the refined models with movements

To examine the distribution of the residues selected as alternative conformers from the MD trajectories, the RMSD (root mean square deviation) of the model before and after refinement was calculated using PyMOL.^66^ The root mean square fluctuation (RMSF) of the refined structures was calculated using the following formula:

$$\rho {\text{RMSF}}_{i}=\sqrt{\frac{1}{N}\;\sum_{n=1}^{N}{\left(r_{i,n}-\left\langle r_{i}\right\rangle \right)}^{2}},$$ (1)

where $\rho {\text{RMSF}}_{i}$ is the RMSF of atoms *i* and $r_{i,n}$ is the coordinate of an atom of interest in frame *n,* and *N* is the total number of frames. $\left\langle r_{i}\right\rangle$ is the mean of the coordinates of atom *i* across all *n* frames. The per-residue RMSF was calculated by taking the mean of all atomic-wise RMSFs for each residue

$${\text{RMSF}}_{\text{resi}}=\sqrt{\frac{1}{M}\;\sum_{i=1}^{M}(\rho {{\text{RMSF}}_{i})}^{2}},$$ (2)

where ${\text{RMSF}}_{\text{resi}}$ is the RMSF of the residue and *M* is the number of atoms in the residue. Ideally, the RMSF of the refined structure should be below 1 Å, indicating the stability of the refined coordinates.^69^

### Identification of the structure models with good geometry and density metrics

The crystallographic model had to be optimized to minimize the errors evaluated by both local and global parameters. Local parameters assess specific aspects of individual residues or small regions within the structure by evaluating the geometry of the model, such as bond lengths, bond angles, and torsion angles, as well as the accuracy and precision of electron density fitting. Global parameters, in contrast, involve broader features of the entire structure, such as R-factors, which assess how well the model fits the experimental diffraction data. To identify structural models with a good balance between local accuracy and global consistency, we defined a composite score for the models obtained by refinement of each MD frame,

$$\text{Composite}\;\text{score}=R_{\text{factor}}+\text{Geometry}\;\text{score}+\text{Density}\;\text{score}.$$ (3)

The composite score consisted of three main terms, each capturing different structural quality aspects. The first term is the summation of R-factors,

$$R_{\text{factor}}=R_{\text{free}}^{\prime }+R_{\text{work}}^{\prime }.$$ (4)

Here, these metrics are individually normalized using min-max scaling, where the minimum and maximum values are determined across all of the models under consideration, ensuring that all variables are rescaled within the range of zero to one. This transformation prevents metrics with inherently large values from disproportionately influencing composite scores,

$$S\prime =\frac{S-\text{min}\left(S\right)}{\text{max}\left(S\right)-\text{min}\left(S\right)}.$$ (5)

Geometry score accounts for structural metrics, MolProbity score, Ramachandran outlier, rotamer outlier, C_β_ outlier, RMS_bond_ outliers, and RMS_angle_ outliers, Clash score, and RMSD of conformer B with respect to A. MolProbity score uses a logarithmically weighted combination of geometric parameters,^49^ which provides a comprehensive and statistically robust evaluation of stereochemical accuracy. Other outlier quantifiers were also used to penalize frames with high outliers while calculating composite scores. This ensures that the composite score reflects both the individual geometric features and their combined quality, thereby enhancing the overall reliability of the evaluation. The RMSD of conformer B relative to conformer A was included as a parameter to filter out structures with a high deviation. These scores are also normalized using min-max scaling, Eq. (5), and summed as

$$\mathrm{Geometry score}=\text{MolProbity}\prime +\text{Ramachandran}\prime +\text{Rotamer}\prime +{C\prime }_{\beta }+{\text{RMS}\prime }_{\text{bond}}+{\text{RMS}\prime }_{\text{angle}}+\text{Clash}\prime +\text{RMSD}\prime .$$ (6)

Density score comprises of terms: RSCC, RSZO, RSZD+, and RSZD−. In the isomorphous difference density map, the residual contribution associated with the difference density was not uniform, with certain residues contributing more than others. To emphasize the residues with greater contributions to the estimation of the RSZD score, the per-residue RSZD was first calculated, and their values were weighted based on the involvement of each residue in the isomorphous difference density map, which is defined as the sum of the difference density contributions for selected residues calculated using ResiDEM.^70^ These weights were multiplied with RSZD+ and RSZD− for positive and negative density, respectively. The weighted RSZD was thus made as a combined score from the summation of the absolute value of RSZD+ and RSZD− given by the following equation:

$${\text{RSZD}}_{n}=\left(\frac{1}{m}\sum_{j=1}^{m}\rho_{\text{negative},j}\left|{\text{RSZD}}_{j,n}^{-}\right|+\rho_{\text{positive},j}\left|{\text{RSZD}}_{j,n}^{+}\right|\right),$$ (7)

where *n* denotes a particular sampled frame of interest and the score is obtained by the summation of RSZD electron density contributions across *m* residues that exhibit difference density features using $\rho_{\text{negative},j}$ and $\rho_{\text{positive},j}$, electron density sum for the negative and positive difference density feature of residue *j* in isomorphous difference density map identified from ResiDEM, respectively, as weights. It should be noted that the RSZD score was calculated using (m*F*o-D*F*c) difference map.

The residue-level metrics RSCC and RSZO were considered in the composite score by taking the average of the residue-level metric values for each frame

$${\bar{v}}_{n}=\left(\frac{1}{R}\sum_{r=1}^{R}v_{n,r}\right),$$ (8)

where *v_n_*_,__*r*_ denotes the value of the metric, RSCC or RSZO, for residue *r* in frame *n*, and *R* is the total number of residues with alternative conformers, providing ${\bar{v}}_{n}$ as the average value for a given frame *n*. Following this, the same min-max normalization was applied, and the values of RSCC and RSZO were inverted by subtracting them from 1, as higher values for the RSCC and RSZO density metrics denote better correlation:

$$\text{Density}\;\text{score}=\text{RSZD}\prime +\left(1-\text{RSCC}\prime \right)+\left(1-\text{RSZO}\prime \right).$$ (9)

## RESULTS

### Challenges in modeling conformations for triggered-state experimental data

**FIG. 2.**
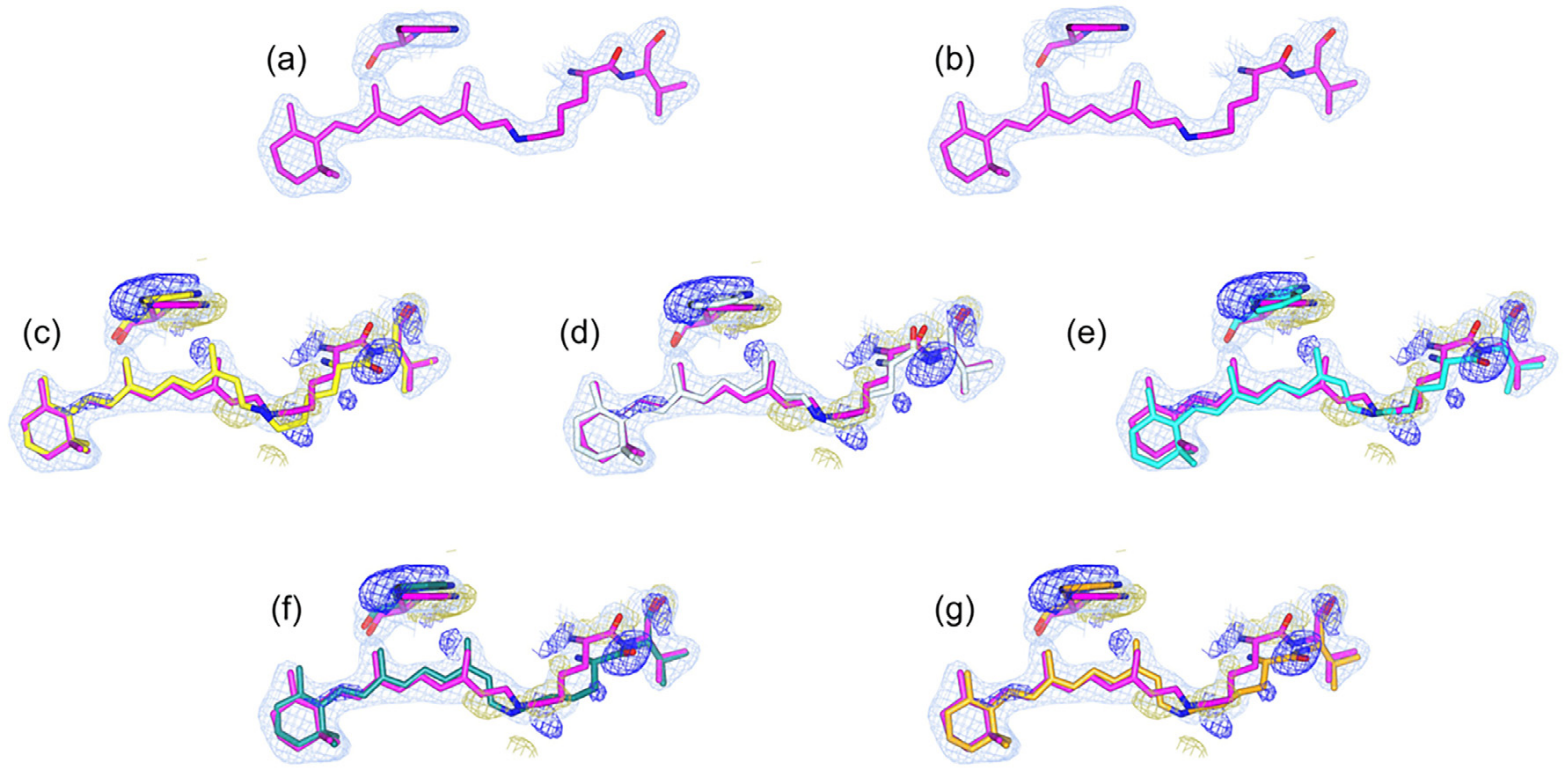
The figure illustrates residues Trp-182, Lys-216, and Ret-300 in bacteriorhodopsin (bR) and their refined models overlaid on 2m*F*o-D*F*c and isomorphous difference density maps. (a) The 2m*F*o-D*F*c density of the reference structure (PDB ID: 5B6V) is shown with the reference structure. (b) The 2m*Fo*-D*Fc* density of the triggered-state data at the 760 ns time point (PDB ID: 5B6X) with the reference structure. (c) The superposition of the triggered-state 2m*F*o-D*F*c density with the isomorphous difference density map on the reference structure emphasizes the difference density features. A triggered-state structure modeled (yellow) within the 2m*F*o-D*F*c density demonstrates the correlation with features observed in the isomorphous difference density map. (d) A triggered-state structure derived from refinement, starting with the dark-state structure, is represented as white sticks. (e) The triggered-state conformer with the lowest R-free is represented as cyan sticks. (f) The triggered-state conformer with the lowest R-work is depicted as teal sticks, and (g) the triggered-state conformer with the lowest composite score is shown as light orange sticks. The 2m*F*o-D*F*c density is represented as a light sky-blue mesh contoured at 1*σ*, and the positive and negative densities in the isomorphous difference density map are depicted as blue and gold meshes, respectively, contoured at 3*σ*. The density maps were clipped to a 2.0 Å radius for clarity. For the calculation of the 2m*F*o-D*F*c density, the respective PDB files were used for the structure factor (*F*c) and phase calculations. Overlays of all refined structures are provided as supplementary material Fig. S1.

The 2m*F*o-D*F*c density alone is often insufficient to accurately position the atomic model in real space because of the subtlety of the conformational shifts and low occupancy of the triggered state observed in the original study of bR. For instance, Fig. 2(a) shows the 2m*F*o-D*F*c density for the resting state (PDB ID: 5B6V), and Fig. 2(b) shows the density of the triggered state at the 760 ns time point (PDB ID: 5B6X) with the resting structure. Both of the maps appear nearly identical, making it difficult to distinguish between the reference- and triggered-state conformations based solely on their densities. In order to address this issue, the isomorphous difference density is often overlaid onto a triggered-state map (2m*F*o-D*F*c) to model the conformations of the triggered-state structure [Fig. 2(c)]. The isomorphous difference density feature highlights the regions of conformational change, revealing the direction of movement between states. The published refined structure of the triggered state (PDB ID: 5B6X), which aligned with the difference in density, is shown in Fig. 2(c).

As an initial test to assess the quality of the model obtained using *phenix.refine* with the employed refinement protocol, the triggered-state structure was refined using the reference-state structure as the starting model.^27^ When the refined reference-state structure was fitted to the 2m*F*o-D*F*c electron density map of the triggered state, it did not fully satisfy the isomorphous difference density [Fig. 2(d)]. Thus, manual adjustments of the B-conformer are required for better alignment with the electron density. In addition, the clash scores of these models were higher than those of the reference structure.

### Use of MD-sampled conformations for modeling triggered state

The advent of large-scale computing facilities has made it possible to use MD simulations to analyze the conformational dynamics involved in biochemical processes. The complexity of the triggered-state dynamics, particularly at later time points in the reaction, involves a potentially large number of residues undergoing conformational changes, making transition state modeling increasingly difficult.^9^ The use of sample structures from MD trajectories is an effective approach to address this challenge. Therefore, the objective of this study was to evaluate whether structures sampled from MD trajectories can be used to produce accurate models of the triggered state from TR-SFX experimental data and to develop an automated workflow. MD simulations capture molecular motion and generate a range of potential conformations; however, additional protocols are necessary to identify those that most closely coincide with experimental observations. This protocol involves refining the structures of the triggered state of bR using samples derived from MD trajectories and identifying models with crystallographic data quality metrics. Using this approach, we can more effectively resolve alternative conformations in regions where the difference in density indicates movement, with improved accuracy.

To investigate the proposed approach of combining structure refinement with MD sampling, we examined the specific structural placement of residues in the active site around Ret-300. In particular, we focused on the movement of the carbonyl tail of the Lys 216 residue's terminal peptide bond. Figure 2(c) illustrates the placement of the trigger state conformer in the published structure (PDB ID: 5B6X) overlaid with the reference (resting) state. For reference data, we refined the triggered-state structure using a dark (reference) state structure as the initial mode [Fig. 2(d)]. The resulting model revealed inaccuracies in the placement of the Schiff base and backbone carbonyl group, as indicated by their proximity to the negative density on the isomorphous difference density map. Although the carbonyl group was not fully covered in the 2m*F*o-D*F*c map, the dominant positive-density feature in the isomorphous difference density maps suggested a potential terminal flip, as illustrated in Fig. 2(c). This observation highlights that the refinement of the dark-state structure alone is insufficient for accurately modeling this region. However, refinement of the dark-state structure is sufficient for Trp182, where the placement aligns well with the observed densities.

To conduct the proposed structure modeling approach, the frames from MD were first aligned with the Cα atoms of the reference-state structure (PDB ID: 5B6V). The resulting RMSDs of nonhydrogen atoms are below 2 Å for all the frames in the trajectory. The conformations of the resting state and moving residues were combined and used as the initial model for subsequent structural refinements (see Materials and Methods section).

Figures 2(e) and 2(f) show the structures of the 760 ns models with the lowest R-free and R-work, respectively, overlaid with the isomorphous difference map, while Fig. 2(g) denotes the structure associated with the model with the lowest composite score (discussed in detail later). Supplementary material Table S1 reports MolProbity validation statistics for the refined bR models. In these models, the B-conformer was positioned away from the negative density and toward the positive density, particularly for the carbonyl tail of the terminal peptide bond of Lys 216, indicating better correlations with the isomorphous difference density map. This shift in the carbonyl tail could also accommodate a positional change in the Schiff base, moving away from the negative density. Both play crucial roles in regard to proton transfer and are covalently connected to the light-absorbing retinal of the bR, facilitating trans-cis interconversion. In the initial frame of the MD trajectory, the carbonyl group was located near the dark reference structure. During the MD trajectory, a subset of frames aligns with the positive-density feature, occupying its corresponding position resulting from the terminal flip.

To systematically identify the models that align with experimental data, it is necessary to establish selection criteria. In crystallography, model validation typically relies on two types of metrics: geometric and electron density metrics, which were validated for each time state and are plotted in Fig. 3 for the 760 ns model and supplementary material Figs. S2–S14 for other time points.

**FIG. 3.**
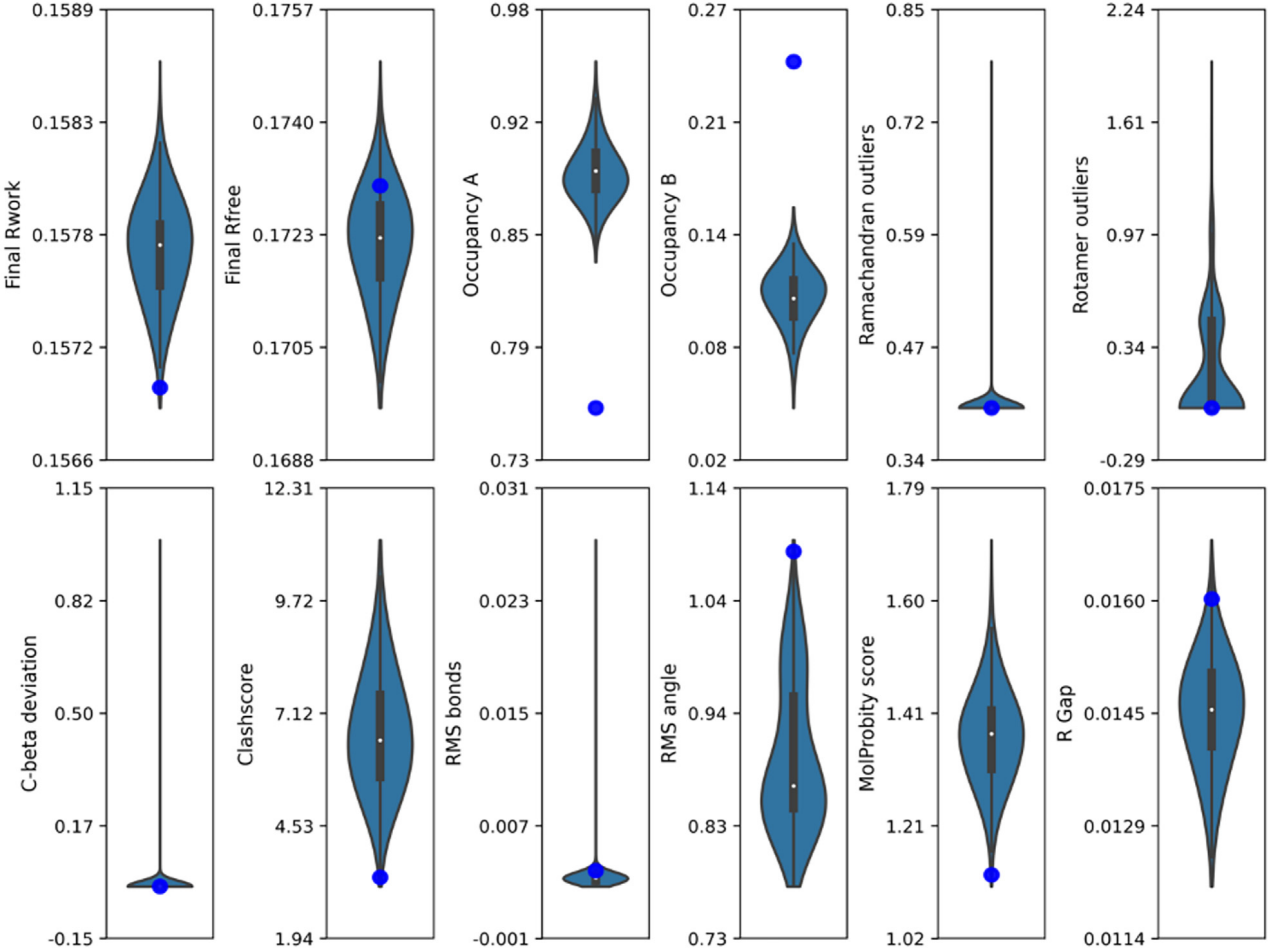
Distribution of the different quality metrics for the structural models derived from refinements of the samples collected through MD trajectories for the 760 ns triggered-state structure. Each violin plot integrates a box plot, where the central marker indicates the median value, and the box length represents the interquartile range (IQR), covering the 25th to 75th percentile of the data. Whiskers extend from the box to depict the data range 1.5 times the IQR from the quartiles. The blue dots represent the corresponding values measured for the published structure (PDB ID 5B6X).

The R-free values of the major portions of the models were found to be lower than those of the published models (Fig. 3). This indicated that the refinement process incorporating MD-sampled conformers can improve the agreement between the model and experimental data. Additionally, R_gap_ (R_free –_ R_work_) tended to increase if the model was overfitted,^20^ but most of the MD-sampled models exhibited a lower R_gap_ than the published models (Fig. 3).

However, a large portion of the MD-derived models had higher clash scores than the published structure (Fig. 3). This suggests that there is room for local model improvement to further reduce steric clashes, ideally to achieve a clash score of zero. However, models with lower clash scores were found to be similar to the reference-state structure, especially around the Schiff base and the carbonyl position of Lys 216. This observation strongly suggests that additional parameters emphasizing the electron density scores must be considered.

**FIG. 4.**
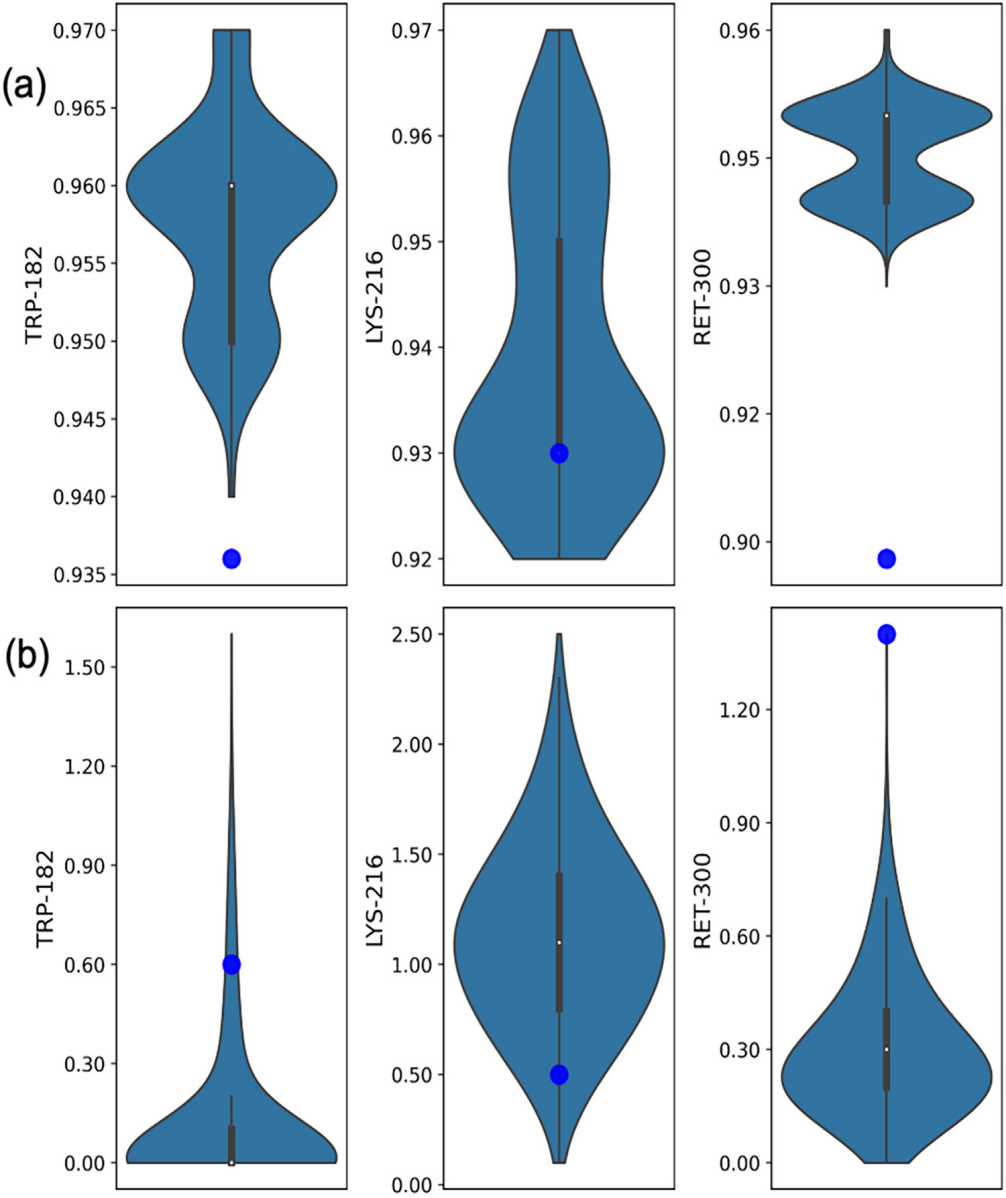
Distribution of the electron density scores obtained from refined frames sampled from MD trajectories. (a) RSCC and (b) RSZD scores for the active-site residues Trp-182, Lys-216, and Ret-300. Each violin plot integrates a box plot, where the central marker indicates the median value, and the box length represents the interquartile range (IQR), covering the 25th to 75th percentile of the data. The whiskers extend from the box to depict the data range 1.5 times the IQR from the quartiles. The blue dots represent the corresponding values measured for the published structure (PDB ID 5B6X).

The residual distributions of the electron density metrics such as RSCC, RSZD, RSZD+, RSZD−, and RSZO for 760 ns are plotted in supplementary material Figs. S15–S19. The RSCC and RSZD for active-site residues such as Trp 182, Lys 216, and Ret 300 are shown in Fig. 4. In many cases, the real-space correlation coefficients (RSCC) of individual residues for the newly derived models using the MD trajectory were better than those of the published structures [Fig. 4(a), supplementary material Fig. S15], highlighting the improvement through this approach with a better overall fit of the electron density. However, a superior RSCC alone does not fully quantify the quality of a model, because it does not account for the weak difference in density associated with the triggered conformational changes. Other electron density metrics, such as the real-space Z-difference score (RSZD), which measures the difference in density in specific regions, showed mixed results [Fig. 4(b), supplementary material Figs. S16–S18]. Certain residues derived from the MD trajectory exhibited very favorable (low) RSZD scores, whereas manually fitted published models performed better than others.

These variabilities clearly show that not all of the conformations from MD sampling are suitable as initial starting models for refinement to build a model associated with the triggered-state diffraction data. Diffraction data were collected at specific time points and the resulting structural model was interpreted as the average of the conformational ensemble at each time point. However, the trajectory of the MD simulation was employed solely as a tool to generate samples of possible conformations, with no intention of generating the conformational ensemble at a specific time point. Therefore, the quality of the models obtained from the integration of MD and structural refinement is expected to be mixed. Nevertheless, the ability to generate high-quality models through exploratory refinement trials is promising.

Because our approach produces a large number of refined models, we must establish a scoring scheme to select frames to improve the chances of identifying the optimal structure. Because the scoring mechanism can be complex owing to the variability in electron density metrics, no single parameter can comprehensively validate a crystallographic model. Thus, we propose a composite score that combines multiple metrics to provide a balanced criterion for selecting the most probable structure from thousands of refined models in the sampled MD frames (see Materials and Methods section).

The model with the lowest composite score fitted well with the electron density, while maintaining consistency with the difference density features [Fig. 2(g)]. The R-free value of the model was slightly lower than that of the published model (supplementary material Fig. S20), thus indicating improved data agreement.

In terms of accuracy based on the electron density, the model also exhibited a comparable overall RSZD, suggesting a similar accuracy based on the electron density. For example, for 760 ns data, the sum of RSZD scores for non-water residues of the model with the lowest composite score was 8.7, compared to 7.1 for the published model, 7.8 for the frame with the lowest R-work, and 9.2 for the frame with the lowest R-free. The clash scores for the models were 4.19, 3.35, 5.24, and 7.13 for the lowest composite score model, the published model, the frame with the lowest R-work, and the frame with the lowest R-free, respectively. These comparisons indicate that the weighted composite score effectively captures the models that balance the electron density fit and geometric quality. Although R-free or R-work values can be examined to identify suitable models, they may require manual review and refinement to resolve clashes. In contrast, the composite score provides a convenient method for selecting suitable candidate models. Overall, the results supported the use of MD sampling to identify models optimized across both local and global structural features.

**FIG. 5.**
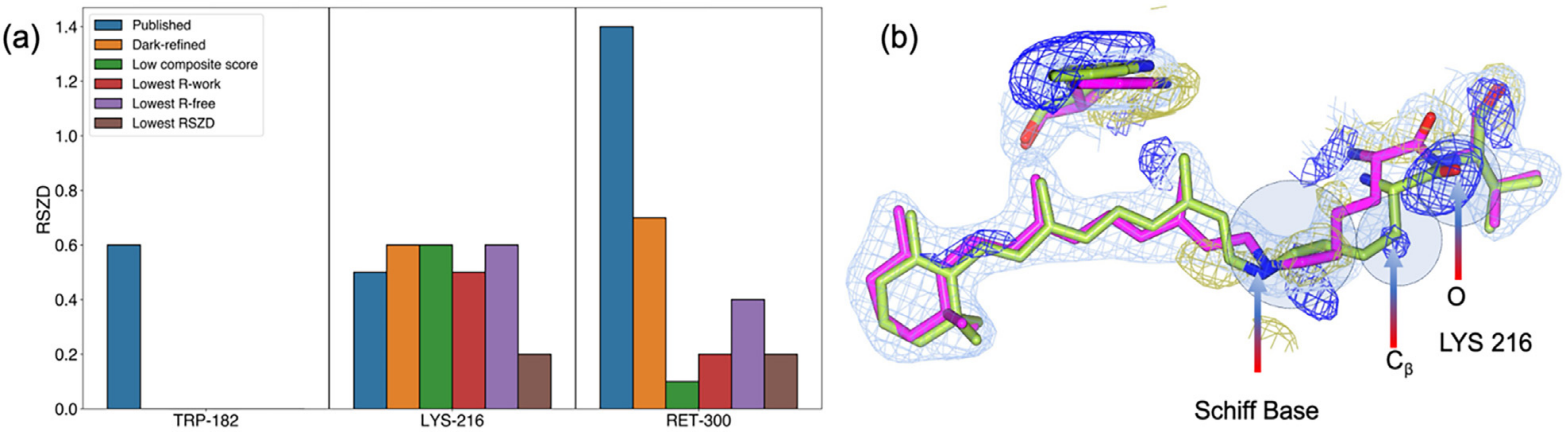
(a) The RSZD score plot for the active-site residues Trp-182, Lys-216, and Ret-300 across the different models, color-coded as published, dark-refined, lowest composite score, lowest R-work, lowest R-free, and lowest RSZD. Here the model with the lowest RSZD is specific for the model where Lys-216 has the lowest RSZD score. These scores provide insight into the fit of the model to local electron density, with an acceptable RSZD score typically being below 3. (b) Image representing the superposition of the triggered-state model (green sticks) with the lowest Lys-216 RSZD score refined from MD-based samples for the 760 ns along with reference structure (green stick). These models are enclosed within the 2m*F*o-D*F*c density shown as a light sky-blue mesh, contoured at 1.0*σ*, with the superposed isomorphous difference density map shown as blue (positive) and gold (negative) mesh, contoured at 3.0*σ.*

We further evaluated the effectiveness of the composite score in selecting the best-fit models by analyzing key active-site residues such as Trp 182, Lys 216, and Ret 300. The structure selected based on the composite score exhibited overall lower RSZD scores for the residues Trp 182 and Ret 300, compared to the published structure [Fig. 5(a)]: around 0.6 and 2.4 for the published structure, and 0 and 0.1 for the model with the lowest composite score, respectively. This finding confirms that the proposed approach effectively identifies structures with greater accuracy. Conversely, the RSZD score for Lys 216 was found to be higher in the model selected using the composite score. To evaluate the models, we specifically examined the one with the lowest Lys 216 RSZD [Fig. 5(b)] and found that it was more accurately aligned with the positive difference density feature in the isomorphous difference density near Lys 216. This alignment was particularly evident for the carbonyl flip and the overlap of positive density around the Lys 216 Cβ atom. The model with the lowest Lys216 RSZD score could effectively capture the minute shift in Cβ atom. However, the RSZD scores of all the models were below 0.6, which is well below the acceptable score of 3. Thus, all the models sufficiently captured the positive difference density associated with carbonyl flip and movement of Cβ toward the positive difference density feature.

We have applied our approach to obtain refined models at other time points in the TR-SFX data to examine if the proposed MD-based approach produces consistent results. Overall, the MD-based models generally exhibited final R-free values that were better than those of the published model, and the R-work values were comparable to those of the published models across all time points (supplementary material Figs. S2–S14). For the models at later time points, where more residues contributed to the isomorphous difference density, clashes associated with the alternative conformer increased. These clashes were primarily due to the short hydrogen contacts between the atoms in the triggered-state model and those in the reference-state structures in neighboring regions. Supplementary material Figs. S20–S30 show the collective overview of geometry and refinement validation metrics (Ramachandran statistics, rotamer outliers, Cβ deviations, clashscore, bond and angle RMSDs, MolProbity score, resolution, R-work, and R-free) across all 13 time points.

We also noted that estimations of occupancy using MD-based conformers showed a wider range when compared to published data for the 760 ns triggered structure. The values obtained from the MD-based approach range from 4% to 16%, whereas the published occupancy rate is 24%. This variation could arise from differences in atomic positions, which influence how the occupancy of the conformer is determined and may reflect changes in structural representation.

**FIG. 6.**
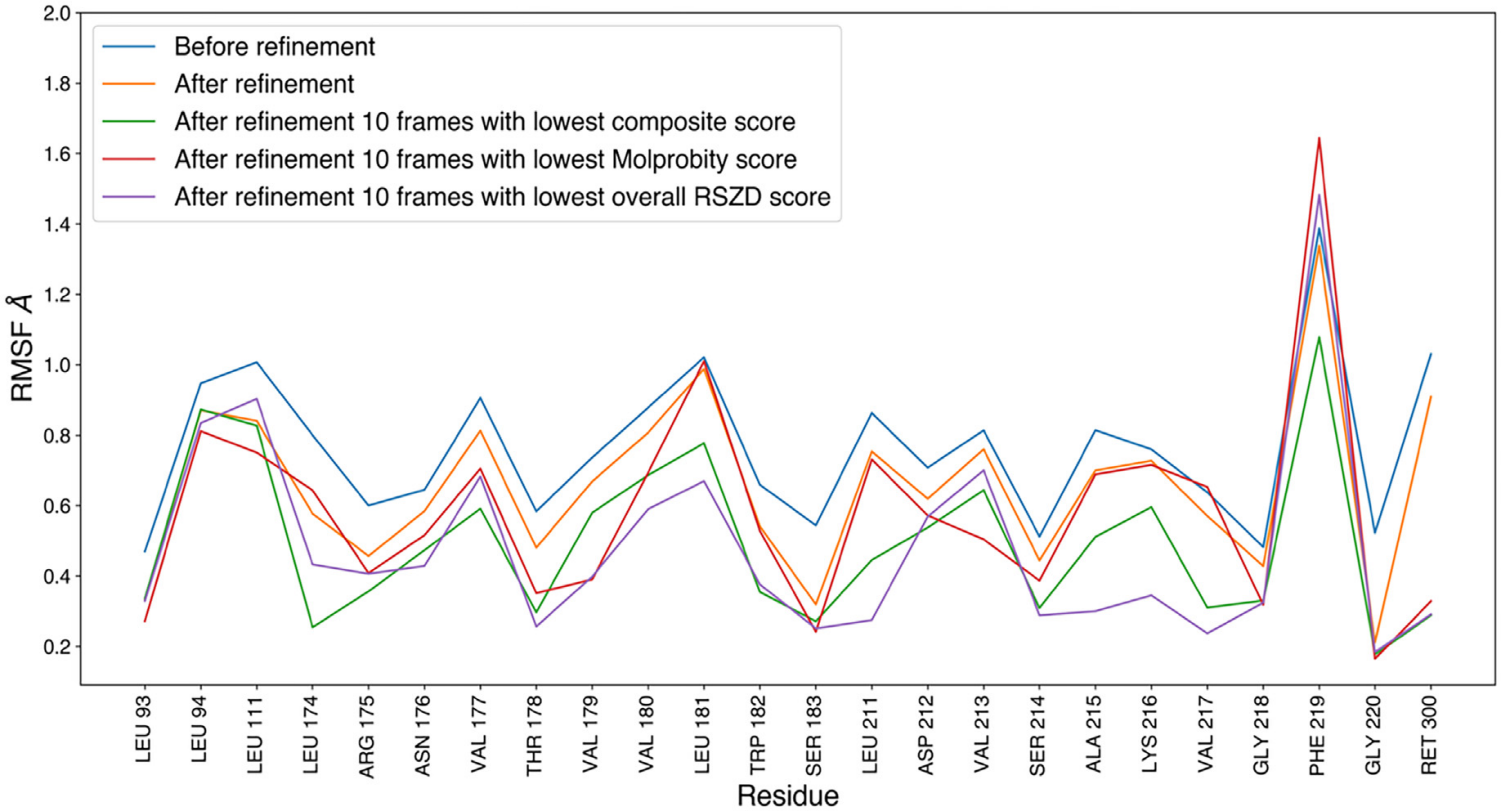
Residue-wise RMSF of the input structures taken from the MD frames before the refinement, and the RMSF of the structures after the refinement against the 760 ns data. RMSFs for the 10 models with the lowest composite scores, lowest MolProbity scores, and lowest RSZD scores, are also plotted. The inset image displays the RMSF of atoms after refinement, colored proportionally to their values.

**FIG. 7.**
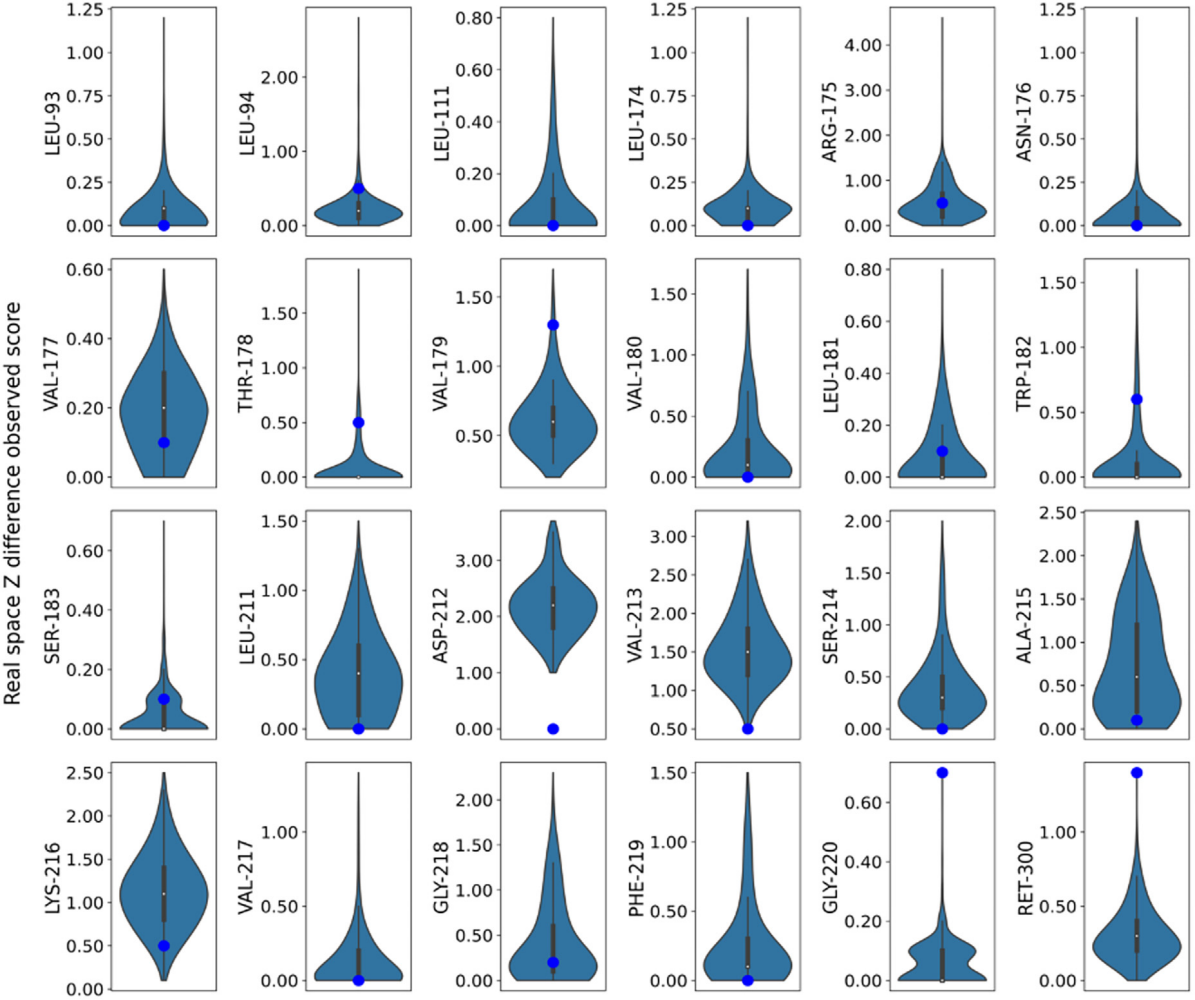
Distribution of RSZD scores for residue in 760 ns triggered-state structure refined from samples obtained via MD trajectories, presented as a violin plot. The blue dot represents the RSZD score for the published structure.

In addition to assessing refinement accuracy, it is also important to consider the structural heterogeneity captured by MD sampling. MD-derived samples help reveal a range of possible conformations, unlike static structures that capture only a single state. Using structures from MD frames allows the sampling of transient states associated with subtle structural transitions. A wide range of input models was used as starting points for refinement. The RMSF plots of the MD frames before refinement reflected the heterogeneity of the atomic positions of the residues captured in the triggered-state MD simulation, with the residues exhibiting different degrees of movement (Fig. 6). The models obtained after refinement still showed large variations, and the heterogeneity observed in the MD frames remained in the refined model. However, not all refined models can be considered good-quality models. Therefore, we additionally analyzed the RMSF of the 10 frames with the lowest composite score, MolProbity score, and RSZD score, all of which can be considered good models based on the experimental data. On average, the RMSFs for the structures with composite scores were lower than those for the models with MolProbity or overall RSZD scores. Nevertheless, heterogeneity was preserved even in frames with the lowest composite, MolProbity, and RSZD scores. This is further validated in Fig. 7, which shows that the RSZD scores of the residues were below three, indicating a well-representative model. Refined conformers for these residues with the lowest scores, together with the corresponding 2*F*o–*F*c, *F*o–*F*c, and *F*o–*F*o maps, are shown in supplementary material Fig. S31. In the case of Phe 219, which had the highest RMSF (Fig. 6), its RSZD distribution showed most of its values closer to zero (Fig. 7), indicating that the diverse models of Phe 219 were all consistent with the experimental data and that the heterogeneity in the models observed in the RMSF cannot be rejected from the RSZD score data. Although the ultimate goal is to determine an accurate structure that meets the electron density criteria for the atomic position, occupancy, and B-factor, this analysis indicates that the observed model variations are consistent with the experimental data.

## DISCUSSION

The primary goal of TR-SFX is to visualize changes in proteins and track molecular movements over time, particularly during chemical reactions, to observe dynamic processes. The ultimate objective of this field is to combine snapshots of transient states and provide a quasi-continuous view of protein dynamics. However, this technique poses significant challenges for data processing.^9^ Structural transitions are often subtle, and refinement of the state structure within the triggered-state density often fails to capture the subtle nuances of the triggered-state structure, as refinement attempts tend to return a structure similar to the reference state owing to its higher population.^9^ Although multiconformational model refinement is feasible for regular crystallographic densities, these methods have not been widely applied to assess their effectiveness in sampling TR-SFX data.

In TR-SFX, conformational shifts can be positional or involve changes around dihedral angles or rotameric states, making accurate assessment of the triggered structure essential for deriving dynamic information. The generation of a multiconformational model refinement is possible with tools such as Ringer,^19^ FLEXR^20^ (which uses Ringer to automate the procedures), and qFit3^21^ designed to sample the electron density and generate associated multiconformers. Ringer and FLEXR sample electron density around the dihedral angles of the protein side chains but do not accommodate backbone shifts. qFit3, on the other hand, generates many both main- and side-chain conformations and computes an occupancy-weighted set of conformations.

We experimented with FLEXR and qFit3 to examine the feasibility of multiconformer modeling for TR-SFX data. Although both tools are effective for many applications, their workflows are designed to consider the entire model and electron density for refinement, which makes it challenging to focus specifically on the dynamic residues associated with the isomorphous difference density. Because this approach did not highlight residues in these regions, we chose not to pursue further testing for this particular dataset.

We have shown that MD trajectories serve as an effective starting point for understanding how specific conformations are associated with the structural and functional states of proteins. By simulating dynamic interactions at the atomic level, MD provides insights into the underlying forces, such as hydrogen bonding, van der Waals interactions, and electrostatic contributions, that stabilize a particular conformation of the residues. A well-positioned initial structure is crucial for modeling triggered data with minor conformational changes, and MD-derived data serve as a reliable starting point for refinement. The RMSF of the refined structure highlights the regions corresponding to signals (electron density) from alternative conformers, where MD-based sampling can effectively capture and elucidate conformational heterogeneity.

Thus, MD-based sampling is a promising approach for refining structural models by exploring a wider range of conformations and potentially reducing the R-free value of the final model. The R-free values of the most refined models generated through MD sampling were lower than those of the published structures, reflecting an improved global fit to the experimental data. Local electron density metrics such as the real-space correlation coefficient (RSCC) and real-space difference Z-score (RSZD) are also critical for evaluating the precision of individual residues. RSCC measures how well the model agrees with the electron density, whereas RSZD indicates the degree of deviation between the model and experimental data. Frames identified from the MD conformations consistently exhibited a higher RSCC than the manually fitted models, demonstrating better agreement with the electron density maps. Most MD-based refinements showed reduced RSZD scores for many residues, indicating improved accuracy in capturing the triggered-state conformation. Additionally, a composite score was employed to identify high-quality models. This score combines geometric metrics with weighted electron density metrics to ensure the selection of the best model that captures the features of triggered states.

Despite these improvements, some residues refined from MD conformations still display potential for further refinement, and the models are often accompanied by increased clash scores due to steric clashes involving neighboring side-chain atoms. Comparatively, previous studies using tools such as FLEXR reported higher clash scores with their Ringer-based approach. Similarly, multiconformational models generated using qFit3 showed higher clash scores, potentially because of the method's focus on fitting multiple conformers to the overall electron density, rather than emphasizing specific dynamic residues.^20^ qFit3 generates conformer samples based on a single-input structure derived from an experimental electron density map. It uses an iterative algorithm to identify and fit multiple conformations to the electron density by optimizing the placement and occupancy of the alternate conformers. This approach reduces the computational cost compared with methods that simulate conformational dynamics or samples beyond the initial input structure.^20^ The MD-based sampling approach can be automated although refining large MD samples requires a significant amount of time.

Another potential weakness of the proposed approach is the limitation of the MD sampling itself. Owing to computational costs, simple MD simulations cannot sample very large conformational transitions, and if a conformation captured in the TR-SFX data is never sampled during MD, the proposed approach may not be effective. In such cases, enhanced conformational sampling techniques for MD simulation could be employed.^71–74^ In addition, with the decreasing computing cost facilitated by the availability of supercomputers, clusters, accelerators, and multiprocessing, MD-based sampling can be effectively used in order to identify models that resemble the triggered-state structures.

We also envision many future developments to further improve the clash score and evaluation metrics for per-residue RSZD to enhance the accuracy of model identification. An approach that has not been tested in the current implementation is the “residue-level electron density fitting” from MD frames, which involves selecting each residue from the MD frames to achieve the highest RSCC and lowest RSZD scores, thereby identifying an accurate residue-level model. Because such a strategy would increase the computational costs, only the overall composite score combining per-residue measurements for each MD frame was evaluated.

Additionally, modeling of water associated with alternative conformers was not undertaken to avoid the complexities; for example, it requires identifying suitable water molecules in MD frames from the weak difference density peaks and adjustment of refinement parameters accordingly. However, this is an important direction for methodological improvement in future studies, as water molecules often play critical roles in functions by influencing residue motions and chemical reactions. For example, water molecules near the key residues could be selected via a distance cutoff for inclusion in structure refinement while maintaining computational feasibility.

There is also room to address inter-residual clashes. The clashes observed at later time points mainly result from the treatment of alternate conformers during refinement. In our workflow, residues showing difference density were modeled with additional conformers (A and B), while the original ground-state geometry was kept unchanged (A). As a result, residues moved as conformer B may clash with neighboring residues fixed in the ground-state conformation. Such effects become more noticeable when more movable residues are introduced at later time points. Additional refinement iterations or simulated annealing protocol for side chains with clashes could help reduce this issue; however, only three macrocyclic refinements were performed due to computational costs. In addition, the protocol for selecting residues to be modeled with additional conformations could be revised to allow additional coordinate refinement. The main advantage of MD-based sampling is that it can effectively sample both small- and large-scale movements associated with the triggered-state density. With these directions, the approach holds promise for effectively modeling conformational heterogeneity with improved geometry.

## CONCLUSION

TR-SFX aims to unravel complex relationships between protein structure and function. In this study, we demonstrated that refinement of the triggered-state conformation using MD trajectory frames as an initial model can yield models with lower R-free values and improved geometry and electron density metrics, thereby contributing to a more accurate and dynamic understanding of protein structures. The region with a high variability in the pool of refined structural models highlighted the regions corresponding to signals from alternative conformers, where MD-based sampling effectively revealed conformational ambiguities across different timescales. Thus, this study has underscored the value of integrating MD simulations with structure refinement techniques for TR-SFX data analysis to capture the dynamic processes of proteins, paving the way for future innovations in modeling and refinement techniques that could further refine our understanding of protein dynamics and functions.

## SUPPLEMENTARY MATERIAL

See the supplementary material for additional data supporting this study: a validation statistics table for the models and residue quality metrics for 760 ns time point, overlays of refined structures with electron density maps, and quality metrics for structural models for all the time points.

## Data Availability

The data that support the findings of this study are openly available in Zenodo at https://doi.org/10.5281/zenodo.17292145 (Ref. 75). The archive includes the MD trajectory that was used to generate conformation samples, refined structural models with favorable refinement statistics described in this study, corresponding map coefficients, and isomorphous difference maps at different time points, and video morphs of the models over the time point series, as well as the scripts for the workflows.
